# Evaluation of a fully absorbable poly-4-hydroxybutyrate/absorbable barrier composite mesh in a porcine model of ventral hernia repair

**DOI:** 10.1007/s00464-016-5057-9

**Published:** 2016-07-01

**Authors:** Jeffrey R. Scott, Corey R. Deeken, Robert G. Martindale, Michael J. Rosen

**Affiliations:** 1 Department of Molecular Pharmacology, Physiology and Biotechnology, Brown University, 171 Meeting Street, Box G-B, Providence, RI 02912 USA; 2 C. R. Bard, Inc. (Davol), Warwick, RI USA; 3 Covalent Bio, LLC, St. Louis, MO USA; 4 Oregon Health & Science University, Portland, OR USA; 5 Cleveland Clinic, Cleveland, OH USA

**Keywords:** Absorbable, Hernia repair, Mesh, Phasix™, Poly-4-hydroxybutyrate, Biologically derived

## Abstract

**Background:**

The objective of this study was to evaluate the mechanical and histological properties of a fully absorbable poly-4-hydroxybutyrate/absorbable barrier composite mesh (Phasix™ ST) compared to partially absorbable (Ventralight™ ST), fully absorbable (Phasix™), and biologically derived (Strattice™) meshes in a porcine model of ventral hernia repair.

**Methods:**

Bilateral abdominal surgical defects were created in twenty-four Yucatan pigs, repaired with intraperitoneal (Phasix™ ST, Ventralight™ ST) or retromuscular (Phasix™, Strattice™) mesh, and evaluated at 12 and 24 weeks (*n* = 6 mesh/group/time point).

**Results:**

Prior to implantation, Strattice™ demonstrated significantly higher (*p* < 0.001) strength (636.6 ± 192.1 N) compared to Ventralight™ ST (324.3 ± 37.1 N), Phasix™ ST (206.9 ± 11.3 N), and Phasix™ (200.6 ± 25.2 N). At 12 and 24 weeks, mesh/repair strength was significantly greater than NAW (*p* < 0.01 in all cases), and no significant changes in strength were observed for any meshes between 12 and 24 weeks (*p* > 0.05). Phasix™ mesh/repair strength was significantly greater than Strattice™ (*p* < 0.001) at 12 and 24 weeks, and Ventralight™ ST mesh/repair strength was significantly greater than Phasix™ ST mesh (*p* < 0.05) at 24 weeks. At 12 and 24 weeks, Phasix™ ST and Ventralight™ ST were associated with mild inflammation and minimal–mild fibrosis/neovascularization, with no significant differences between groups. At both time points, Phasix™ was associated with minimal–mild inflammation/fibrosis and mild neovascularization. Strattice™ was associated with minimal inflammation/fibrosis, with minimal neovascularization at 12 weeks, which increased to mild by 24 weeks. Strattice™ exhibited significantly less neovascularization than Phasix™ at 12 weeks and significantly greater inflammation at 24 weeks due to remodeling.

**Conclusions:**

Phasix™ ST demonstrated mechanical and histological properties comparable to partially absorbable (Ventralight™ ST) and fully resorbable (Phasix™) meshes at 12 and 24 weeks in this model. Data also suggest that fully absorbable meshes with longer-term resorption profiles may provide improved mechanical and histological properties compared to biologically derived scaffolds.

Ventral hernia repair remains one of the most common and costly general surgery procedures, with an estimated 350,000 repairs performed annually within the USA at a cost of ~$3.2 billion [1]. The field of hernia repair has been revolutionized in the 138 years since Billroth first envisioned the concept of prosthetic hernia repair. In 1878, he stated, “*If we could artificially produce tissues of the density and toughness of fascia and tendon the secret of the radical cure of hernia would be discovered.*” [2] In the early 1900s, metal meshes were introduced, followed by synthetic polymer meshes in the 1950s [2]. Since that time, the field has experienced significant biomaterial advancements, which have led to the development of a wide variety of prosthetics, including permanent synthetic polymers, biologically derived bioprosthetics, and absorbable synthetic/biosynthetic polymers.

Permanent synthetic polymer meshes provide long-term mechanical support to the hernia defect and have been shown to reduce recurrence rates compared to suture repair without mesh [3, 4]. However, these permanent materials have also been associated with a chronic inflammation [5–7] and may be susceptible to seeding of the mesh or erosion into bowel and subsequent chronic bacterial colonization [8–15]. Biologically derived bioprosthetics were expected to at least partially overcome these issues, due to their biological origin and ability to be remodeled and revascularized. However, this remains a controversial point as recent studies have demonstrated that biologically derived prosthetics may also be susceptible to the negative effects of bacterial colonization, resulting in prolonged colonization and premature degradation [8, 16–18]. Studies have also shown that some biologically derived prosthetics exhibit inconsistent properties due to variation in donor tissue characteristics and quality control [19]. Furthermore, a minority of patients may also have religious, cultural, or ethical concerns regarding the use of animal- or human-derived products [20]. Taken together, absorbable synthetic and biosynthetic polymers that largely do not have these concerns may represent an attractive alternative. These materials are not derived from mammalian sources and can be produced with established textile manufacturing techniques and possess uniform characteristics with predictable degradation profiles.

A variety of absorbable synthetic/biosynthetic meshes are now available including microporous sheets and macroporous multifilament or monofilament meshes. The first absorbable synthetic meshes were comprised of multifilament woven materials, such as Vicryl^®^ mesh (Ethicon, Inc., Somerville, NJ) and microporous sheets, such as Gore^®^ BIO-A^®^ Tissue Reinforcement (W.L. Gore & Associates, Inc., Flagstaff, AZ). Macroporous multifilament absorbable synthetic mesh has also been introduced, including TIGR^®^ Matrix Surgical Mesh (Novus Scientific, Uppsala, Sweden). More recently, biosynthetic meshes have been generated from biologically derived, non-mammalian sources, including multifilament SERI^®^ Surgical Scaffold (Allergan, Inc., Irvine, CA) derived from silkworms and monofilament Phasix™ mesh (C. R. Bard, Inc./Davol, Inc., Warwick, RI) with raw material (poly-4-hydroxybutyrate, P4HB) derived and subsequently purified from a cellular source (genetically modified K12 *E. coli* bacteria).

An established absorbable mesh barrier technology already in wide clinical use (Sepra^®^) has recently been paired with Phasix™ mesh (load-bearing scaffold) to create the first fully absorbable composite mesh (Phasix™ ST mesh) for soft tissue reconstruction. The Sepra^®^ barrier technology is a hydrogel that has been optimized to swell upon rehydration to reduce the development of peritoneal tissue attachments to the underlying mesh. Preclinical studies have previously demonstrated the efficacy of this mesh coating absorbable barrier technology [21–24].

The objective of this study was to evaluate the mechanical and histological properties of a fully absorbable poly-4-hydroxybutyrate/absorbable barrier composite mesh (Phasix™ ST) compared to partially absorbable (Ventralight™ ST), fully absorbable (Phasix™), and biologically derived dermal matrix (Strattice™) meshes in a porcine model of ventral hernia repair.

## Methods

### Study compliance

This study was reviewed by the Institutional Animal Care and Use Committee (IACUC) of CBSET, Inc. (Lexington, MA), and was conducted in compliance with all regulations regarding humane treatment of laboratory animals.

### Study design

Twenty-four (*n* = 24) female, juvenile, Yucatan swine (41.5–51.5 kg, 8–10 months old at implantation) were acquired for the study and randomly assigned to one of the four mesh groups (*n* = 6 animals per mesh type). Half of the animals in each group were assigned to a 12-week survival period (*n* = 3 animals, *n* = 6 repairs per mesh type), while the other half were assigned to a 24-week survival period (*n* = 3 animals, *n* = 6 repairs per mesh type). The four groups of meshes included: Phasix™ ST, Ventralight™ ST, Phasix™, and Strattice™ meshes. All four mesh devices evaluated are commercially available and have received 510(k) clearance by the FDA for indications that include soft tissue repair/reinforcement procedures. The surgical, veterinary, and pathology aspects of this study were conducted by trained study-site personnel.

### Meshes evaluated

**Fig. 1:**
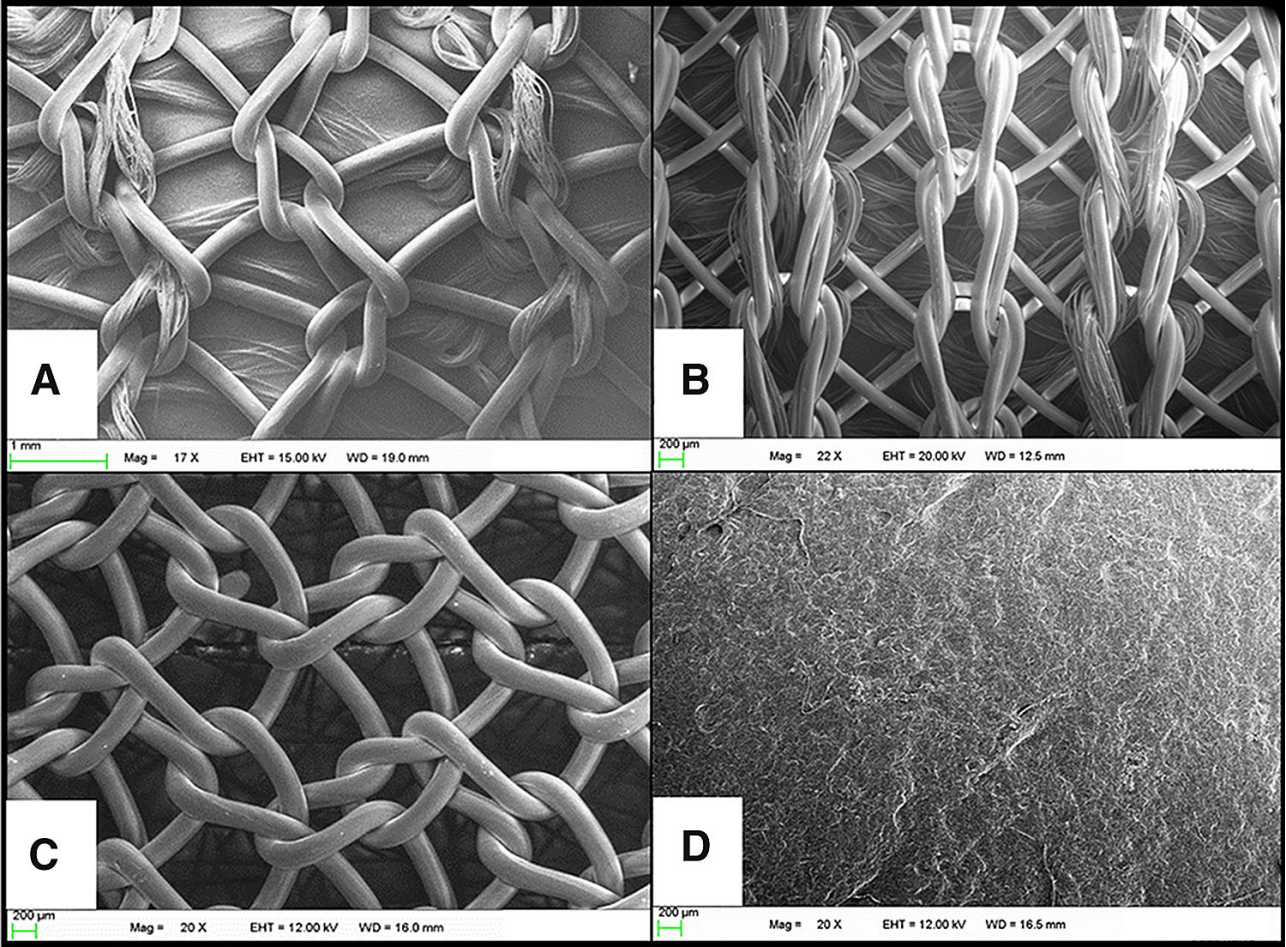
Scanning electron micrographs of meshes prior to implantation (T_0_): **A** Phasix™ ST (17 × magnification), **B** Ventralight™ ST (22 × magnification), **C** Phasix™ (20 × magnification), and **D** Strattice™ meshes (20 × magnification)

As shown in Fig. 1, Phasix™ ST (C. R. Bard, Inc./Davol Inc., Warwick, RI) is a macroporous, fully absorbable, composite mesh device that consists of co-knitted absorbable poly-4-hydroxybutyrate (P4HB) and polyglycolic acid (PGA) fibers coated with a chemically modified sodium hyaluronate (HA), carboxymethylcellulose (CMC), and polyethylene glycol (PEG)-based hydrogel on the visceral surface. Ventralight™ ST (C. R. Bard, Inc./Davol Inc., Warwick, RI) is a macroporous, partially absorbable, composite mesh device that consists of co-knitted permanent polypropylene and absorbable polyglycolic acid (PGA) fibers coated with a HA/CMC/PEG-based hydrogel on the visceral surface. The primary load-bearing component of the device is non-absorbable (polypropylene). Phasix™ (C. R. Bard, Inc./Davol Inc., Warwick, RI) is a macroporous, fully absorbable mesh device that consists of knitted absorbable monofilament poly-4-hydroxybutyrate fibers. Strattice™ (LifeCell Corp., Branchburg, NJ) is a biologically derived prosthetic comprised of acellular, non-cross-linked porcine dermis.

### Perioperative preparation of animals

The animals were not offered their daily food ration prior to surgical induction, but water was not restricted. On the morning of surgery, Rimadyl^®^ (Carprofen: 2.2 mg/kg, PO) and Telazol^®^ (4-6 mg/kg IM) were administered. Isoflurane anesthesia was then administered to effect until the animals were in a plane of anesthesia that facilitated endotracheal intubation. Animals were intubated and maintained with isoflurane to effect for the remainder of the surgical procedure. An IV catheter was placed in a peripheral ear vein for administration of supportive IV fluids. Buprenorphine (0.02 mg/kg, IM) was administered, along with preemptive antibiotic therapy [ceftiofur (5 mg/kg, IM) and excede (5 mg/kg, IM)]. Animals were placed in dorsal recumbent position, and the abdomen was shaved and prepared for aseptic surgery using accepted veterinary care standards.

### Intraperitoneal (underlay) mesh placement

A 30-cm midline incision was created, and a midline laparotomy performed to open the peritoneal space. Two lateral 1-inch (2.54 cm) surgical defects were created, one on each side of the midline, using a circular die-cutting tool. The tool was rotated to create a full-thickness muscular defect through the lateral and transverse oblique muscles. The muscle at the defect site was then reapproximated with 2-0 PDS™ suture, and the peritoneum was closed over the repaired muscular defect using 2-0 PDS™ suture. Phasix™ ST and Ventralight™ ST meshes were die-cut into 3.25-inch (8.26 cm)-diameter circles and hydrated in sterile saline for 1–3 s. Meshes were then fixated to the peritoneum over the defect site using 12–14 SorbaFix™ fasteners around the periphery of the mesh. A single mesh type was implanted in a given animal. The abdomen was closed using standard closure techniques. Bupivacaine was infused into the midline incision site (not exceeding 2 mg/kg). Analgesics were administered for the first 72 postoperative hours and continued on an as-needed basis thereafter [buprenorphine (0.02 mg/kg) and Rimadyl^®^ (2.2 mg/kg, PO)]. Animals were survived for 12 or 24 weeks (*n* = 3 animals for each mesh type per time point) and then sedated with Telazol^®^ (4–6 mg/kg, IM), anesthetized with isoflurane, and euthanized via an overdose of potassium chloride solution, IV.

### Retromuscular (sublay) mesh placement

Animals with meshes implanted in the retromuscular plane underwent an identical surgical protocol as those with meshes placed intraperitoneally, except that the peritoneum was left intact in these animals and was therefore not incised or repaired. Briefly, two lateral 1-inch (2.54 cm) surgical defects were created, one on each side of the midline, using a circular die-cutting tool. The tool was rotated to create a full-thickness muscular defect through the lateral and transverse oblique muscles. The muscle at the defect site was then reapproximated with 2-0 PDS™ suture, and Phasix™ or Strattice™ was implanted in the retromuscular plane and fixated circumferentially with 12 SorbaFix™ fasteners (for Phasix™) or 12 Nurolon™ sutures (for Strattice™), respectively.

### Mechanical testing

**Fig. 2:**
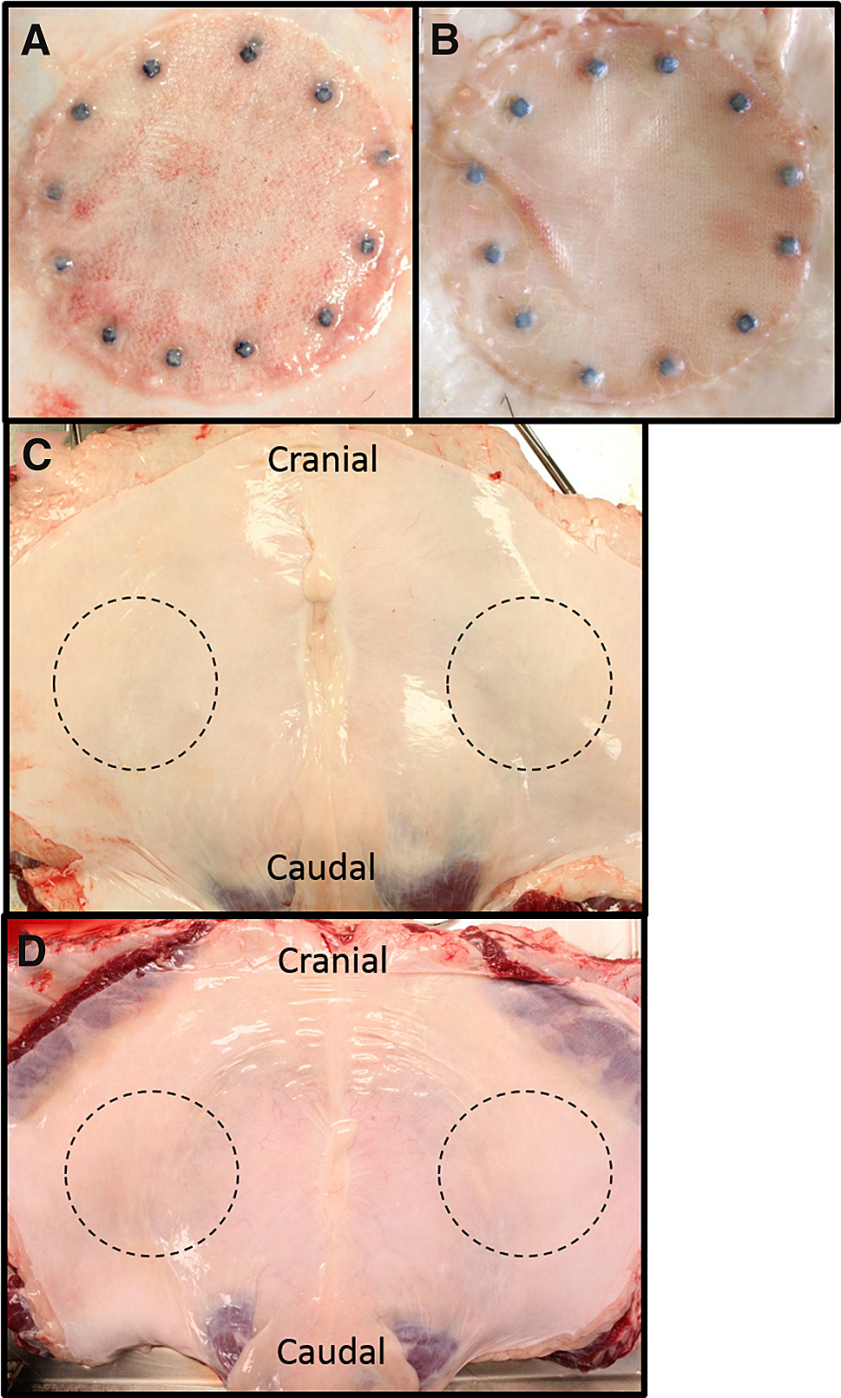
Gross necropsy photographs of mesh at 24 weeks postimplantation: **A** Phasix™ ST, **B** Ventralight™ ST, **C** Phasix™, and **D** Strattice™ meshes

Following euthanasia, as shown in Fig. 2 the abdominal skin was dissected from the cranial portion of the abdomen, and the entire abdominal wall (including the two surgical defects repaired with mesh and the native abdominal wall (NAW) tissue immediately lateral to the mesh) was excised and placed in a non-permeable plastic transport bag with small amount of saline solution (0.9 % sodium chloride). All mechanical evaluations were accomplished at Altran Solutions (Boston, MA) within 24 h of explantation using a universal electromechanical testing system (Instron^®^, MTS) with a 2kN load cell and a data acquisition rate of 10 Hz. Non-implanted meshes were hydrated and evaluated to establish the baseline (T_0_) ball burst strength of each mesh type (*n* = 8 Strattice™ and *n* = 6 Phasix™, Phasix™ ST, and Ventralight™ ST). Specimens of corresponding NAW (without mesh or hernia defect) were also obtained immediately lateral to the defects and mechanically evaluated to establish the baseline characteristics of the porcine abdominal wall in the absence of a hernia defect or mesh repair (n = 6 for each time point/mesh group). The entire mesh/repair site (3.25 inch diameter) was evaluated for the explanted meshes (n = 6 for each time point/mesh group). All Nurolon™ sutures and SorbaFix™ fixation devices were left intact within the mesh/repair site, and the entire specimen including fixation devices was clamped within the test fixture. All mechanical evaluations were conducted on explanted specimens that were clamped inbound of fixation points, thereby eliminating the impact of fixation on mechanical testing. Specimens measured 3.25 inches in diameter and were mounted in a test fixture between two polymer plates measuring 12 × 16 inches each and coated with 120-grit sandpaper to maximize grip and prevent slippage of the tissue. The upper plate had an aperture of 0.43 inch diameter to accommodate passage of the 0.37-inch-diameter probe and an aperture of 0.87 inch diameter in the lower plate to avoid unnecessary stress on the tissue as it was displaced by the probe. The 0.37-inch-diameter probe was centered directly over the defect site in each specimen and applied in compression at a rate of 1 inch/min until it burst through. The peak load was recorded as the ball burst strength (N). The NAW strength was calculated as the average of specimens obtained from animals in the 12- and 24-week groups combined.

### Histological analysis

Following mechanical testing, the 3.25-inch-diameter mesh/repair specimen was cut in half, lengthwise through the defect, and half of the specimen was placed in 10 % neutral buffered formalin for histological analysis. The specimens were then processed, embedded, sectioned at ~5 µm, and stained with hematoxylin & eosin (H&E) and Masson’s trichrome (MT). Slides were prepared from areas lateral to the repair rather than centered over the defect area of the specimen. Histological evaluation was conducted by an independent, board-certified veterinary pathologist at CBSET, Inc. (Lexington, MA). The host tissue response was assessed via microscopic evaluation of H&E-/MT-stained slides. The host inflammatory/fibrotic response and neovascularization were scored for one section of each slide according to an established semiquantitative scale: 0 = no response; 1 = minimal/barely detectable; 2 = mild/slightly detectable; 3 = moderate/easily detectable; and 4 = marked/very evident [23–25].

### Statistical analysis

Data were collected, analyzed, interpreted, and graphically displayed using GraphPad Prism 6.01 statistical software. Baseline mesh strength values (T_0_) were compared via a one-way analysis of variance (ANOVA), followed by Tukey’s multiple comparison post test. A one-way ANOVA was also performed on the postimplantation ball burst data, followed by Bonferroni’s multiple comparison post test. Data are presented as mean ± standard deviation. A nonparametric Kruskal–Wallis analysis was performed on the histology data (0–4 point scale), followed by Dunn’s multiple comparison post test. Data are presented as median with interquartile range (25-75 %). The threshold of statistical significance was set at *p* < 0.05. Meshes implanted in the intraperitoneal and retromuscular planes were analyzed separately.

## Results

### Mechanical testing

**Fig. 3:**
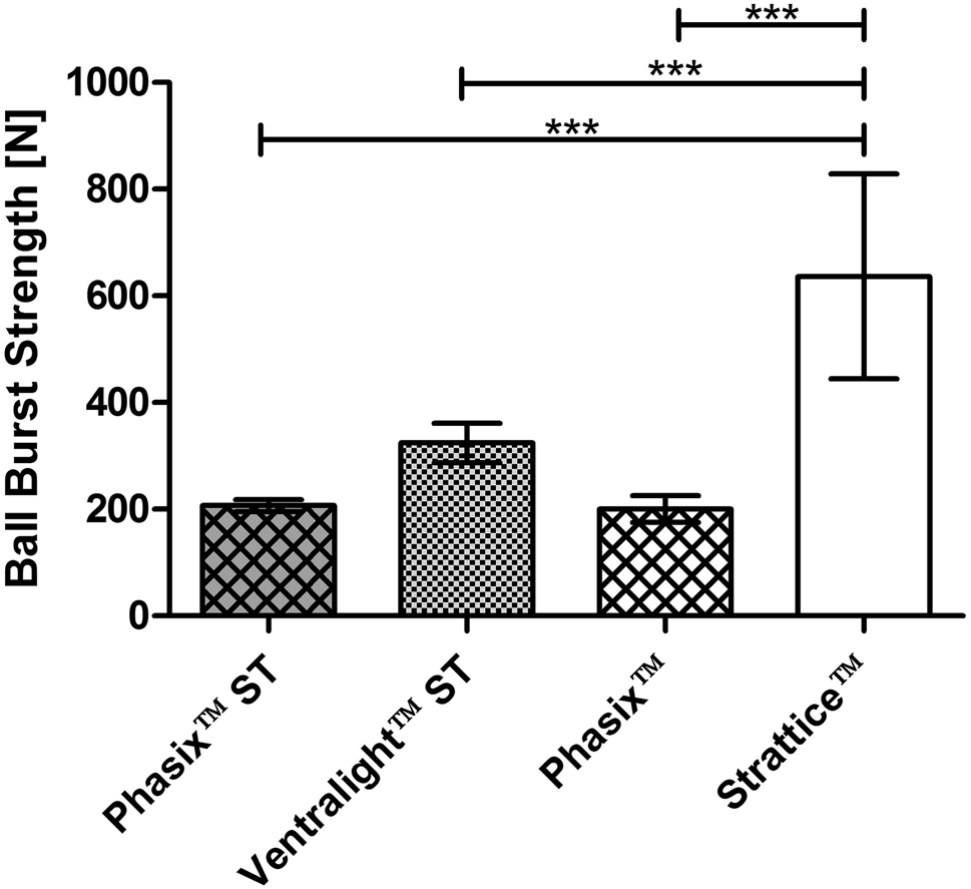
Ball burst strengths of Phasix™ ST (*n* = 6), Ventralight™ ST (*n* = 6), Phasix™ (*n* = 6), and Strattice™ (*n* = 8) meshes prior to implantation (T_0_). Data presented as mean ± standard deviation (***p < 0.001)

As shown in Fig. 3, the hydrated preimplantation strength (T_0_) of Strattice™ (636.6 ± 192.1 N) was significantly greater than the hydrated preimplantation strength of Ventralight™ ST (324.3 ± 37.1 N), Phasix™ ST (206.9 ± 11.3 N), and Phasix™ (200.6 ± 25.2 N) (*p* < 0.001 in all cases when tested in a hydrated state).

### Intraperitoneal (underlay) mesh placement

**Fig. 4:**
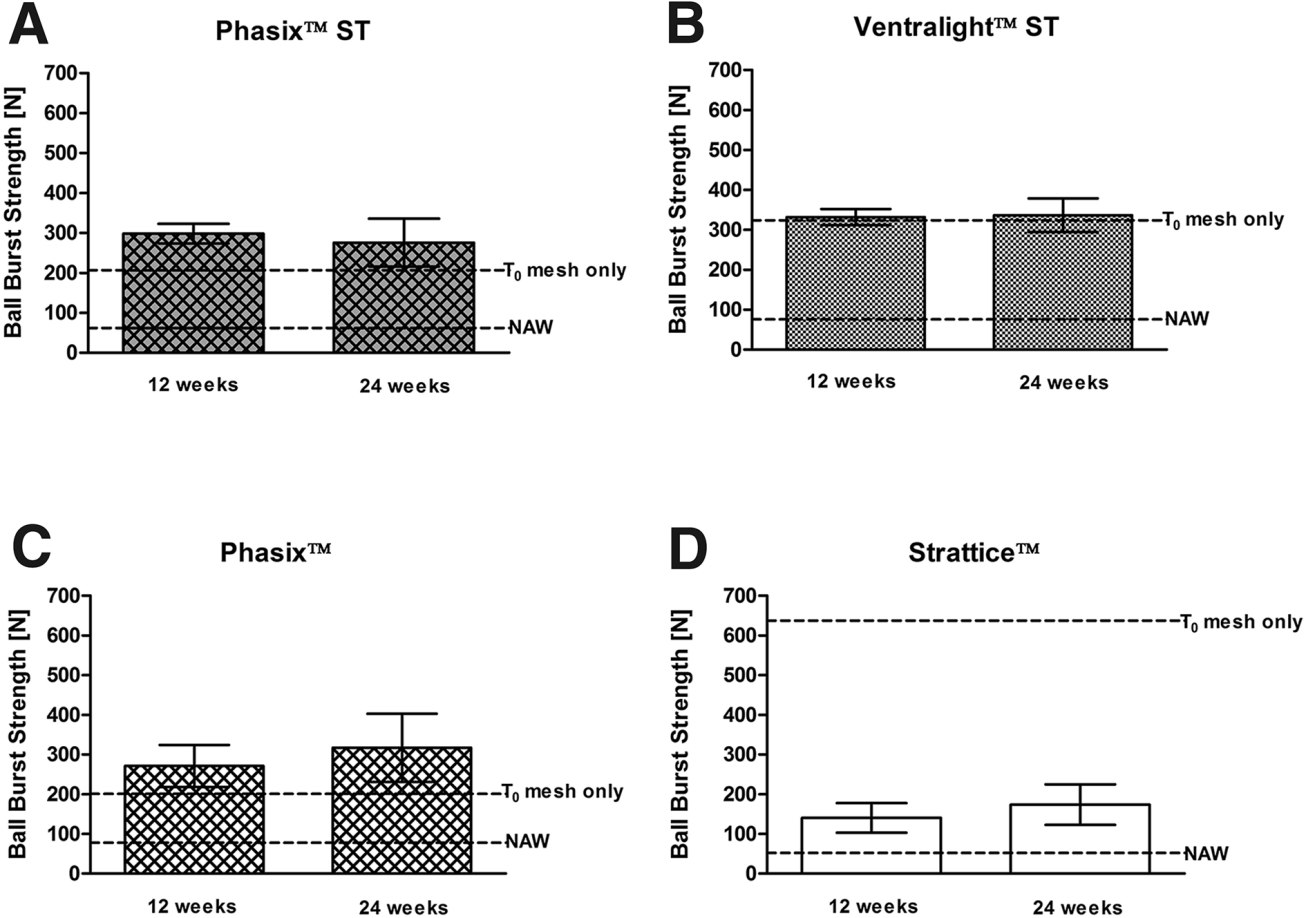
Mesh/repair strengths at 12 and 24 weeks postimplantation (*n* = 6 of each mesh type per time point): **A** Phasix™ ST, **B** Ventralight™ ST, **C** Phasix™, and **D** Strattice™ meshes. Data presented as mean ± standard deviation. Mean ball burst strengths of meshes prior to implantation (T_0_) and NAW presented as *dotted lines*

As shown in Fig. 4A, the Phasix™ ST mesh/repair strength at 12 weeks (298.4 ± 24.7 N) was 44 % greater than the T_0_ strength of the mesh alone. Furthermore, the Phasix™ ST mesh/repair strength at 24 weeks (275.8 ± 59.6 N) was 33 % greater than the T_0_ strength of the mesh alone. However, it should be noted that this increase in strength relative to the T_0_ strength of the mesh alone represents the contribution of the abdominal wall, any newly formed tissue, and the mesh scaffold. No significant change in mesh/repair strength was observed for Phasix™ ST between 12 and 24 weeks (−8 %, *p* > 0.05). The mesh/repair strength of Phasix™ ST was also significantly greater than the NAW (61.5 ± 16.7 N) at both 12 and 24 weeks (*p* < 0.001 in both cases).

In contrast, Fig. 4B demonstrates that the mesh/repair strength of Ventralight™ ST at 12 weeks (331.9 ± 20.2 N) and 24 weeks (336.4 ± 42.1 N) remained similar to the T_0_ strength of Ventralight™ ST alone. Additionally, no significant change in mesh/repair strength was observed for Ventralight™ ST between 12 and 24 weeks (+1 %, *p* > 0.05). However, the mesh/repair strength of Ventralight™ ST was significantly greater than the NAW (76.0 ± 27.6 N) at both 12 and 24 weeks (*p* < 0.001 in both cases).

**Fig. 5:**
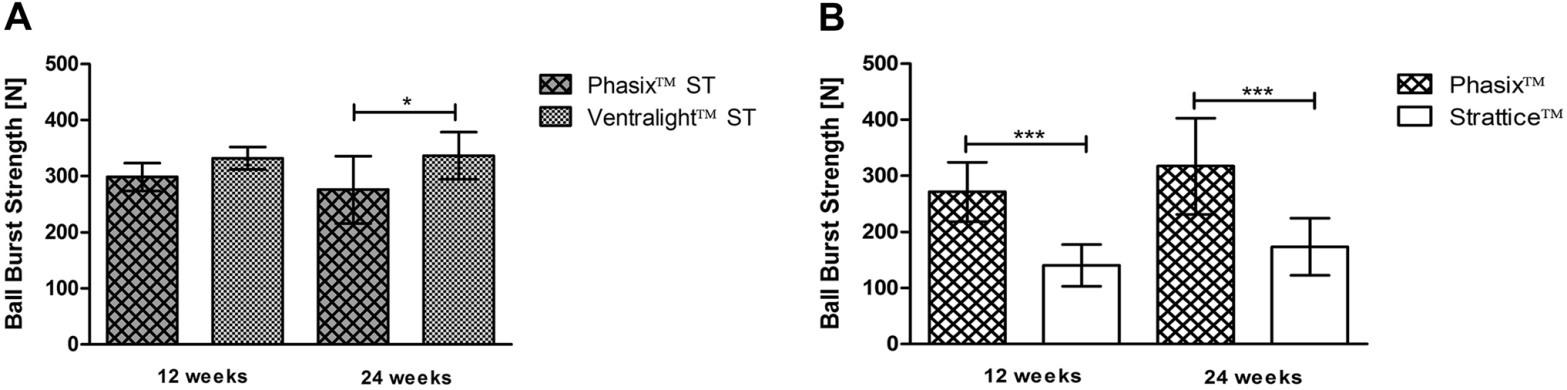
Ball burst strengths of mesh-repaired sites at 12 and 24 weeks postimplantation (*n* = 6 of each mesh type per time point): **A** Intraperitoneal plane (Phasix™ ST and Ventralight™ ST meshes) and **B** retromuscular plane (Phasix™ and Strattice™ meshes). Data presented as mean ± standard deviation (*p < 0.0; ***p < 0.001)

As shown in Fig. 5A, no significant difference in mesh/repair strength was observed between Phasix™ ST (298.4 ± 24.7 N) and Ventralight™ ST (331.9 ± 20.2 N) mesh-repaired sites at 12 weeks (*p* > 0.05). However, the mesh/repair strength of Ventralight™ ST (336.4 ± 42.1 N) was significantly greater than Phasix™ ST (275.8 ± 59.6 N) at 24 weeks (*p* < 0.05).

### Retromuscular (sublay) mesh placement

As shown in Fig. 4C, the Phasix™ mesh/repair strength at 12 weeks (271.4 ± 52.7 N) was 35 % greater than the T_0_ strength of the mesh alone. Furthermore, the Phasix™ mesh/repair strength at 24 weeks (317.1 ± 85.6 N) was 58 % greater than the T_0_ strength of the mesh alone. Again, it is important to note that this increase in strength relative to the T_0_ strength of the mesh alone represents the contribution of the abdominal wall, any newly formed tissue, and the mesh scaffold. No significant change in mesh/repair strength was observed for Phasix™ between 12 and 24 weeks (+17 %, *p* > 0.05). However, mesh/repair strength of Phasix™ was significantly greater than the NAW (78.4 ± 25.0 N) at 12 and 24 weeks (*p* < 0.001 in both cases).

In contrast, Fig. 4D demonstrates that Strattice™ mesh/repair strength decreased by 78 % after implantation for 12 weeks (140.4 ± 37.3 N) and by 73 % after implantation for 24 weeks (173.6 ± 51.1 N) compared to the T_0_ strength of the mesh alone. However, no significant change in mesh/repair strength was observed for Strattice™ between 12 and 24 weeks (+23 %, *p* > 0.05). The mesh/repair strength of Strattice™ was significantly greater than the NAW (51.9 ± 20.2 N) at 12 and 24 weeks (*p* < 0.01 and *p* < 0.001, respectively).

As shown in Fig. 5B, the mesh/repair strength of Phasix™ (271.4 ± 52.7 N) was significantly greater than Strattice™ (140.4 ± 37.3 N) at 12 weeks (*p* < 0.001). Similarly, the mesh/repair strength of Phasix™ (317.1 ± 85.6 N) was significantly greater than Strattice™ (173.6 ± 51.1 N) at 24 weeks (*p* < 0.001).

Although Phasix™ ST versus Strattice™ and Phasix™ versus Ventralight™ ST were not directly compared in the statistical analyses due to implantation in different tissue planes (i.e., intraperitoneal versus retromuscular), some interesting observations were made when reviewing the data for all meshes without regard to tissue plane. At 12 weeks, Ventralight™ ST (331.9 ± 20.2 N), Phasix™ ST (298.4 ± 24.7 N), and Phasix™ (271.4 ± 52.7 N) all displayed similar mesh/repair strength, which were approximately twice the strength exhibited by Strattice™ (140.4 ± 37.3 N). This trend continued at 24 weeks, with Ventralight™ ST ™ (336.4 ± 42.1 N), Phasix™ ST (275.8 ± 59.6 N), and Phasix™ (317.1 ± 85.6 N) all displaying similar mesh/repair strength, which remained approximately 1.5–2 times the strength of Strattice™ (173.6 ± 51.1 N), respectively.

### Histological analysis

**Fig. 6:**
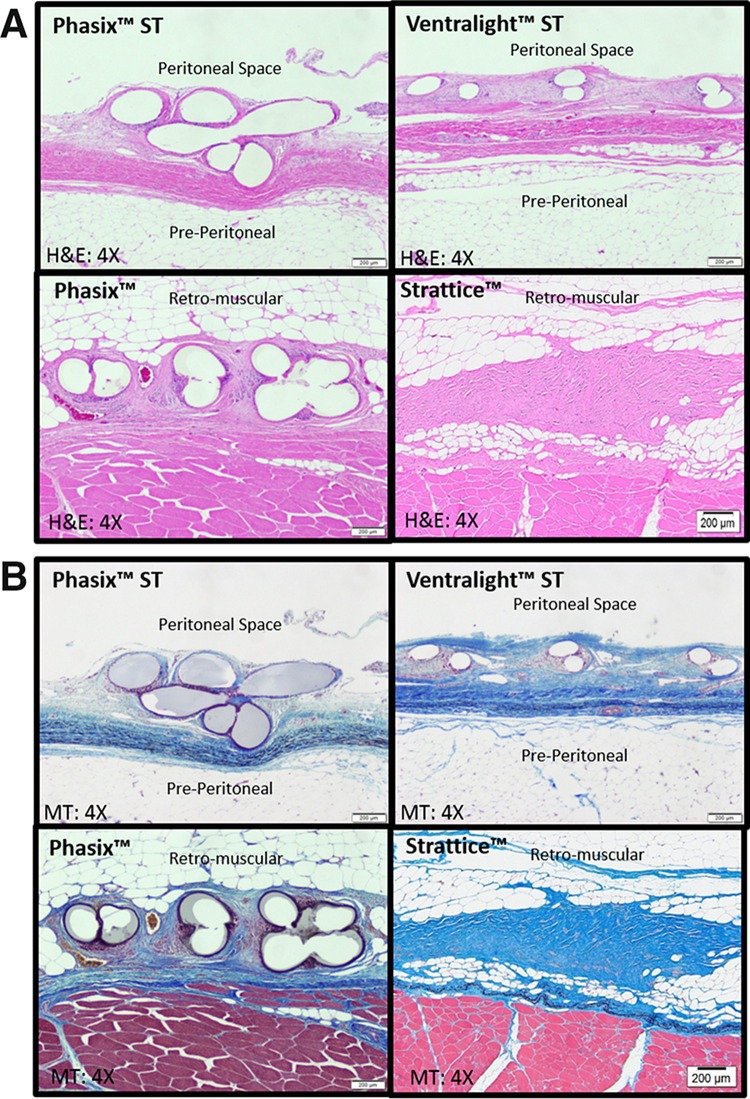
Photomicrographs of Phasix™ ST, Ventralight™ ST, Phasix™, and Strattice™ mesh-repaired sites at 24 weeks postimplantation: **A** hematoxylin & eosin (H&E)-stained slides (4 × magnification) and **B** Masson’s trichrome (MT)-stained slides (4 × magnification)

Figure 6 depicts representative photomicrographs of each mesh type at the time of explantation, which demonstrates a comparable host tissue response regardless of mesh type. Additionally, Table 1 shows similar inflammation, fibrosis, and neovascularization scores for all four mesh types at both of the time points evaluated, although unique differences are documented below.

**Table 1:** Inflammation, fibrosis, and neovascularization scores associated with Phasix™ ST, Ventralight™ ST, Phasix™, and Strattice™ meshes at 12 and 24 weeks postimplantation in a porcine model of ventral hernia repair

Median (interquartile range)	Inflammation score		Fibrosis score		Neovascularization score	
	12 weeks	24 weeks	12 weeks	24 weeks	12 weeks	24 weeks
Phasix™ ST	2.0 (1.0–2.0)	2.0 (1.0–2.0)	1.5 (1.0–2.0)	1.0 (1.0–1.25)	1.0 (0.75–1.25)	1.5 (0.75–2.0)
Ventralight™ ST	2.0 (1.75–2.0)	2.0 (1.75–2.0)	1.5 (1.0–2.0)	1.5 (1.0–2.0)	2.0 (0.75–2.0)	2.0 (1.75–2.0)
Phasix™	1.5 (1.0–2.0)	2.0 (1.75–2.0)	1.5 (1.0–2.0)	1.0 (1.0–2.0)	2.0 (1.75–2.0)	2.0 (1.75–2.0)
Strattice™	1.0 (1.0–1.0)	0.5 (0.0–1.0)***	1.0 (1.0–1.0)	1.0 (1.0–1.25)	1.0 (0.0–1.25)**	2.0 (1.75–2.0)*

Data presented as median (interquartile range)

* Strattice™ meshes demonstrated significantly increased neovascularization between 12 and 24 weeks (*p* < 0.05); ** significantly less neovascularization than Phasix™ at 12 weeks (*p* < 0.05); *** significantly less inflammation than Phasix™ at 24 weeks (*p* < 0.01)

### Intraperitoneal (underlay) mesh placement

As shown in Table 1 and Fig. 6, Phasix™ ST was associated with mild inflammation (median = 2.0) and minimal to mild fibrosis and neovascularization (median = 1.0–1.5) which did not change significantly between 12 and 24 weeks (*p* > 0.05 in all cases). Similarly, Ventralight™ ST was associated with mild inflammation (median = 2.0), minimal to mild fibrosis (median = 1.5), and mild neovascularization (median = 2.0), which did not change significantly between 12 and 24 weeks (*p* > 0.05 in all cases). No significant differences were observed between the inflammation, fibrosis, or neovascularization scores associated with Phasix™ ST and Ventralight™ ST at either 12 or 24 weeks (*p* > 0.05 in all cases). At both time points, the absorbable hydrogel barrier and PGA fibers of Phasix™ ST and Ventralight™ ST were completely resorbed, whereas the primary mesh components of these devices (Phasix™ ST: P4HB, Ventralight™ ST: polypropylene) remained intact.

### Retromuscular (sublay) mesh placement

As shown in Table 1 and Fig. 6, Phasix™ was associated with minimal to mild inflammation and fibrosis (median = 1.0–2.0) and mild neovascularization (median = 2.0) which did not change significantly between 12 and 24 weeks (*p* > 0.05 in all cases). Similarly, Strattice™ was associated with minimal inflammation and fibrosis (median = 0.5–1.0) that did not change significantly between 12 and 24 weeks (*p* > 0.05 in all cases). However, Strattice™ was associated with minimal neovascularization at 12 weeks (median = 1.0 and interquartile range = 0.0–1.25) that significantly increased to mild neovascularization at 24 weeks (median = 2.0 and interquartile range = 1.75–2.0; *p* < 0.05). Furthermore, Strattice™ exhibited significantly less neovascularization at 12 weeks compared to Phasix™ (Phasix™: median = 2.0 and interquartile range = 1.75–2.0 vs. Strattice™: median = 1.0 and interquartile range = 0.0–1.25; *p* < 0.05) and significantly less inflammation at 24 weeks with evident resorption/remodeling of this scaffold compared to Phasix™ (Phasix™: median = 2.0 and interquartile range = 1.75–2.0 versus Strattice™: median = 0.5 and interquartile range = 0.0–1.0; *p* < 0.01). By 24 weeks, the primary mesh component of Phasix™ (P4HB) remained intact, whereas Strattice™ demonstrated evident resorption/remodeling at this time point.

## Discussion

The objective of this study was to evaluate the mechanical and histological properties of a fully absorbable poly-4-hydroxybutyrate/absorbable barrier composite mesh (Phasix™ ST) compared to partially absorbable (Ventralight™ ST), fully absorbable (Phasix™), and biologically derived (Strattice™) meshes in a porcine model of ventral hernia repair.

Mechanical testing revealed that sites repaired with coated (Phasix™ ST) or uncoated (Phasix™) versions of the poly-4-hydroxybutyrate technology had similar mesh/repair strength and were both significantly stronger than NAW at 12 and 24 weeks. These results suggest that Phasix™ ST and Phasix™, with associated tissue ingrowth, augmented the strength of the NAW despite partial resorption of mesh fibers comprising these devices. In addition, Phasix™ ST and Phasix™ demonstrated comparable mesh/repair strength to Ventralight™ ST, which suggests that they may provide medium-term mechanical strength comparable to partially absorbable mesh incorporating a permanent polypropylene scaffold. However, it is noteworthy that Ventralight™ ST exhibited significantly greater mesh/repair strength at 24 weeks compared to Phasix™ ST, which may be due to volume differences in PGA fiber content (Phasix™ ST possesses ~40 % less PGA fiber), biomechanical differences between P4HB/polypropylene load-bearing components, and/or partial resorption of P4HB.

Although exhibiting the greatest preimplantation strength of all biomaterials evaluated in this study, biologically derived (Strattice™) mesh/repairs demonstrated 78 % lower mechanical strength relative to preimplantation strength at 12 weeks, indicating a rapid strength decline in vivo. Comparatively, sites repaired with Strattice™ were significantly weaker than sites repaired with the fully absorbable biosynthetic mesh (Phasix™), as early as 12 weeks. These biologically derived scaffold observations are similar to previously reported results by Cavallo et al. that demonstrated 91 % and 96 % lower mechanical strength for biologically derived (Strattice™) mesh/repairs relative to preimplantation strength at 1 and 6 months, respectively [19, 26]. Similarly, Monteiro et al. also showed a 40, 84, and 81 % reduction in mechanical strength for Strattice™ mesh/repairs relative to preimplantation strength at 2, 4, and 6 weeks, respectively [27]. However, it should be noted that in the current study, the strength of Strattice™ mesh/tissue repairs at both 12 and 24 weeks was still approximately 3 times the strength of NAW alone. Clinical studies are required to determine whether this strength profile is compatible with the dynamic load-bearing requirements of the human abdominal wall in specific targeted patient populations.

As demonstrated through mechanical testing in this porcine study, the relatively slow degradation rate of the load-bearing component within Phasix™ ST and Phasix™ (P4HB) may be advantageous in facilitating a more gradual transfer of load from the mesh back to the native tissue compared to biologically derived or rapidly resorbing synthetic biomaterials. Rapid mechanical degradation of biomaterials could result in premature transfer of load back to the native tissue before the repair site has been fully remodeled and strengthened by mature collagen and may ultimately contribute to hernia recurrence rates. Within the Repair of Infected or Contaminated Hernias (RICH) clinical trial (NCT00617357), Itani et al. previously reported 28 % hernia recurrence at 2 years following ventral hernia repair with biologically derived non-cross-linked porcine dermis (Strattice™) in CDC class II to IV patients [28]. Within the Complex Open Bioabsorbable Reconstruction of the Abdominal Wall (COBRA) clinical trial (NCT01325792), Rosen et al. recently reported 17 % hernia recurrence at 2 years following ventral hernia repair with a synthetic fully absorbable mesh (Gore^®^ Bio-A^®^) in CDC class II to III patients [29]. Additional clinical studies are warranted to determine whether rapid mechanical degradation of the devices evaluated ultimately contributed to the observed hernia recurrence rate and/or whether longer-term fully absorbable biosynthetics could improve upon these results.

The predictable, long-term resorption profile associated with Phasix™ ST and Phasix™ may also be beneficial in protecting these meshes from rapid degradation by collagenases if inadvertently exposed to bacteria during/after implantation. Deeken et al. have shown that Strattice™ is rapidly degraded by collagenases during in vitro studies, leading to a significant loss of mechanical strength, particularly when compared to cross-linked porcine dermis material such as Permacol™ [30]. Additionally, Sahoo et al. have shown that enzymatic degradation of non-cross-linked human acellular dermal matrices leads to a significant decline in mechanical properties and frequently mechanical failure of the material [31]. These results compare well with those of the current study and provide rationale for further evaluation of Phasix™ ST and Phasix™ in future studies with bacterial or collagenase exposure.

Histological analysis of each mesh was performed to provide additional insight into the host response associated with each of the mesh materials evaluated in this study. The results revealed that Phasix™ ST mesh exhibited minimal–mild inflammation, fibrosis, and neovascularization characteristics comparable to Ventralight™ ST and Phasix™. Additionally, these three meshes exhibited similar responses at both 12 and 24 weeks, indicating a stable host tissue response with insignificant changes over time.

In contrast, Strattice™ was associated with significant changes in neovascularization over time. At 12 weeks, neovascularization scores for Strattice™ were significantly lower than scores reported for Phasix™ mesh. However, neovascularization scores for Strattice™ increased significantly between 12 and 24 weeks, reaching levels of neovascularization comparable to the other three mesh types by 24 weeks. Morphological characteristics such as overall surface area may contribute to these temporal differences between materials. The microporous, sheet-like structure inherent in biologically derived scaffolds may slow the overall rate of tissue integration and vascularization compared to the macroporous, monofilament structure of Phasix™ ST and Phasix™, which facilitates rapid integration and neovascularization, similar to traditional and composite ventral hernia repair prosthetics.

Interestingly, Strattice™ was also associated with significantly less inflammation at 24 weeks compared to Phasix™ mesh. This may be explained by the differing resorption profiles associated with these materials. As shown in Fig. 6, Strattice™ was significantly resorbed/remodeled by 24 weeks, while Phasix™ remained largely intact. Thus, the inflammatory response was absent/minimal for Strattice™, but remained an ongoing, mild response for Phasix™.

The results of this study compare well with similar, previously published studies [24, 25, 27, 32]. In 2013, Deeken et al. evaluated Phasix™ in a porcine model of hernia repair at 6, 12, 26, and 52 weeks postimplantation. The results were comparable to the current study and showed that Phasix™-repaired sites were significantly stronger than NAW at all time points with a mild inflammatory response [25]. In another study, Martin et al. evaluated Phasix™ in a porcine model at 8, 16, 32, and 48 weeks postimplantation. Again, the results were comparable to the current study and showed that Phasix™-repaired sites were significantly stronger than NAW at 8 and 16 weeks and comparable to NAW thereafter, with a moderate inflammatory response present at all time points [32]. In a third study, Monteiro et al. evaluated Strattice™ in a porcine model at 2, 4, and 6 weeks postimplantation. These results were also comparable to the results of Strattice™ in the current study and showed a mild inflammatory response with rapid 40, 84, and 81 % decline in mesh strength at 2, 4, and 6 weeks, respectively [27].

There are some limitations associated with the current study that deserve consideration. First, the medium-term implant duration of 24 weeks is limited given the longer-term resorption profile associated with Phasix™ ST and Phasix™ compared to that associated with Strattice™. The results of this study provide an understanding of the short- to medium-term tissue response and mechanical properties associated with these materials. However, future studies should include longer implant durations to more fully characterize the responses associated with these materials after complete resorption of not only the barrier layer, but also the primary load-bearing structural component of the mesh. Additionally, although this study provides important insight using an established large animal model, the impact of mesh resorption on hernia recurrence rates and clinical performance should be addressed in future clinical trials. Furthermore, there are some limitations associated with the T_0_ and NAW specimens chosen for this study. In future studies, it may be useful to also include a T_0_ sample of mesh/tissue repair strength immediately after surgical implantation of the mesh over a repaired defect in addition to the T_0_ mesh-only data acquired here. This type of specimen would provide additional insight into the contribution of the repaired tissue to the strength of the initial mesh/tissue repair prior to mesh resorption or tissue ingrowth. Finally, it should be acknowledged that specimens obtained for histological analysis were taken after the completion of mechanical testing. It is possible that artifacts were created in the tissue specimens due to compression during testing, although no such observations were noted by the pathologist.

## Conclusions

Phasix™ ST demonstrated comparable mechanical and histological properties to partially absorbable (Ventralight™ ST) and fully resorbable (Phasix™) meshes at 12 and 24 weeks in a porcine model of ventral hernia repair. Data also suggest that longer-term, fully absorbable mesh devices may provide more optimal mechanical and histological properties to support gradual load transfer to the abdominal wall than biologically derived scaffolds for soft tissue repair/reconstruction within this porcine model.
